# A Two-Year Follow-Up Cohort Study—Improved Clinical Control over CVD Risk Factors through Weight Loss in Middle-Aged and Older Adults

**DOI:** 10.3390/jcm9092904

**Published:** 2020-09-08

**Authors:** Pawel Macek, Malgorzata Terek-Derszniak, Malgorzata Biskup, Halina Krol, Jolanta Smok-Kalwat, Stanislaw Gozdz, Marek Zak

**Affiliations:** 1 Institute of Health Sciences, Collegium Medicum, Jan Kochanowski University, Zeromskiego 5, 25-369 Kielce, Poland; pawel.macek@onkol.kielce.pl (P.M.); mbiskup@onet.eu (M.B.); krolhalina@poczta.fm (H.K.); stanislawgozdz1@gmail.com (S.G.); 2 Department of Epidemiology and Cancer Control, Holycross Cancer Center, Artwinskiego 3, 25-734 Kielce, Poland; 3 Department of Rehabilitation, Holycross Cancer Center, Artwinskiego 3, 25-734 Kielce, Poland; m.terek@poczta.fm; 4 Research and Education Department, Holycross Cancer Center, Artwinskiego 3, 25-734 Kielce, Poland; 5 Clinical Oncology Clinic, Holycross Cancer Center, Artwinskiego 3, 25-734 Kielce, Poland; jolanta.smok-kalwat@onkol.kielce.pl

**Keywords:** obesity, weight loss, weight maintenance, cardiovascular risk factors, prevention, public health

## Abstract

Modest weight loss enhances clinical control over cardiovascular disease (CVD) risk factors in overweight and obese individuals. This study aimed to assess the associations between individual weight loss and predefined criteria for clinical improvement in blood pressure, lipid levels, and glycemia. A two-year follow-up study involved 3388 (37.9% men) aged 45−64 years, BMI ≥ 25 kg/m^2^. Changes in body weight were calculated as a percentage of baseline weight; outcome variables: systolic (SBP), diastolic (DBP) blood pressure, high-density (HDL-C) and low-density (LDL-C) lipoproteins, fasting blood glucose (FBG), and triglycerides (TG) were construed as the differences between baseline and outcome values. Clinically significant improvement was defined as SBP/DBP reduction by 5 mm/Hg, FBG−20 mg/dL, LDL-C-10 mg/dL, TG−40 mg/dL, and HDL-C increase by 5 mg/dL. Apart from LDL-C, a modest 5%–10% weight loss was associated with clinically significantly improved outcomes. The incident rate ratios and 95% confidence intervals for clinical improvement of SBP were: 1.27 (1.14–1.40), DBP/1.30 (1.12–1.50), HDL/1.54 (1.18–2.02), and TG/1.69 (1.32–2.17). In the higher category of weight loss, associations were still manifest, although the results proved diagnostically challenging (low number of cases). Even though modest weight loss does enhance clinical control over CVD risk factors, offering regular medical guidance to patients is postulated to further boos the anticipated outcomes.

## 1. Introduction

Adult body weight generally changes over time, and these changes appear inevitable. Depending on individual predispositions, over the course of one’s life span, body weight is subject to gradual growth characterised by different dynamics, depending on individual lifestyle, as well as numerous environmental and genetic factors [1]. Body weight gain leading to obesity is a generally acknowledged health hazard, and a major risk factor for cardiovascular diseases (CVDs) [2,3,4,5]. Specific guidelines issued by the American Heart Association/American College of Cardiology/The Obesity Society (AHA/ACC/TOS) unequivocally support the consensus on the health benefits stemming from weight loss [6,7]. Despite diverse methodological approaches, the overall body of research evidence indicates that approx. 5%–10% of total body weight loss accounts for specific health benefits, e.g., lowered blood pressure, reduced cholesterol, and fasting glycemia, although very little is known on actual clinical impact outside this particular scope [8,9,10].

The key objective of treating obesity consists in reducing morbidity and mortality by way of exercising appreciably tighter control over generally acknowledged risk factors for CVDs [11,12]. This notwithstanding, it is still considered, especially with regard to morbidly obese individuals, that planning for weight loss down to the referential value is often unrealistic, or merely produces weight loss of a transient nature. Regaining previous body weight is quite common and, regretfully, burdened with a risk of further deterioration in individual health status [13,14]. Hence, recommending just any amount of weight loss seems a far better strategy, much more manageable in practical terms, easier to maintain, and certain to offer tangible health benefits to any individuals keen to follow its key principles [15].

Even though obesity is commonly associated with an appreciable health risk among most individuals, it does not seem to increase this risk in a small proportion. This phenomenon is referred to as metabolically healthy obesity (MHO) [16]. As this has not been described by a clear definition to date, it is rather hard to estimate its incidence within a population. Some investigators believe that MHO is a transient state, as the long-term (20 years follow-up) studies indicate that at least some of the subjects had become metabolically unhealthy before the conclusion of the study protocol, while some of them were exposed to CVD events in the post follow-up period [4,17,18].

The Polish-Norwegian Study (PONS) conducted in 2010–2011, in association with Polish and Norwegian scientists, offered a unique opportunity to assess the key determinants of health, as well as gain some valuable insights into the actual causes of morbidity and mortality in Poland. The study was a continuation of the International Health Monitoring (HEM) *Closing the Gap* Project, carried out in the Oncology Centre in Warsaw. The study’s location was selected in due consideration of the commonly acknowledged risk paradigms for major non-communicable diseases, exposure to environmental risk factors, level of economic development, identified migration flow patterns, appropriate infrastructure, and long-term commitment to the project’s objectives. Recruitment for the PONS survey was supported by an extensive media campaign. All men and women aged 45–64 years (*n* = 110,000), living in two geographically separate regions, were invited to participate in the PONS survey [19,20,21,22]. Permanent residents were recruited from a single urban district (city of Kielce)—with 60,000 residents, aged 45–64 years, of which 13% were covered by the PONS population sample, and from a single rural district (Kielce province) — with 50,000 residents aged 45–64 years, of which 10% were covered by the PONS population sample. Consequently, within 16 months, 12% (*n* = 13,172) of the target population was recruited for the PONS survey, including 4799 residents of Kielce. After two years, in all participants in the PONS survey (pursued within the framework of the *Healthy Kielce* Survey Project) who were also permanent residents of Kielce (*n* = 4,99) individual health status was reassessed, making use of the same baseline (PONS) methodology [23]. 

The present study aimed to assess the association between the proportion of weight loss and the actual extent of individual health status improvement in terms of blood pressure, lipids level, and fasting glycemia. An attempt was also made to assess the association between the proportion of weight loss and the securing of the predefined criteria for significantly improving clinical control over the risk factors for CVDs.

## 2. Material and Methods

The research data were collected in three different locations by appropriately trained personnel (nurse, interviewer). All participants were examined in line with applicable constraints of the PONS study protocol. Following the receipt of a signed-off, informed written consent form, a biological sample (blood) and pertinent anthropometric measurements were taken, and an interview (Health Status Questionnaire) was conducted. The collected information was entered directly into an online questionnaire, and exported to the data retrieval system on the server, where they were subsequently subjected to a rigorous Quality Assurance procedure, with a view to assessing their overall integrity and screening for any procedural noncompliance. The blood collection and storage procedure were developed in line with applicable standards of Biobanking and Biomolecular Resources Research Infrastructure (BBMRI).

The PONS study was approved by the ethics committee within the Cancer Center and by the Institute of Oncology in Warsaw, Poland. The present study was duly approved by a local Ethics Review Committee, Faculty of Health Sciences (Approval Ref. No. 25), The Jan Kochanowski University (JKU) in Kielce, Poland.

### 2.1. Data Verification

**Figure 1:**
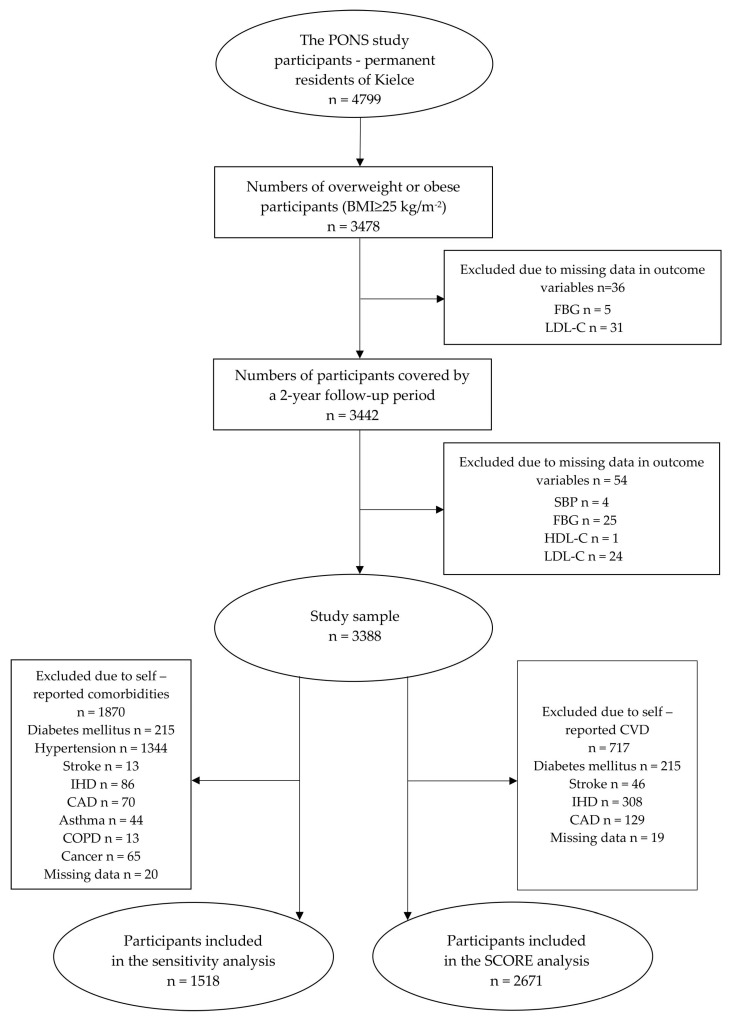
Data selection process in the main and the sensitivity analyses. Abbreviations: SBP, systolic blood pressure; FBG, fasting blood glucose; HDL-C, high-density lipoprotein cholesterol; LDL-C, low-density lipoprotein cholesterol; IHD, ischemic heart disease; CAD, coronary artery disease; COPD, chronic obstructive pulmonary disease; CVD, cardiovascular disease; SCORE, Systematic Coronary Risk Evaluation.

The verification covered the data pertaining to permanent residents of the city of Kielce, participants of the PONS study (*n* = 4799), acquired in 2010–2011. Reassessment of individual health status was carried out in 2012–2013 as part of the *Healthy Kielce* Survey Project. Data of the participants (*n* = 1321) with normal body weight (BMI < 25.0 kg/m^2^) were deleted from the PONS database. This group comprised 18 cases with BMI indicating underweight (<18.5 kg/m^2^). Subsequently, 36 (total) cases of missing data on fasting blood glucose (FBG) and low-density lipoprotein cholesterol (LDL-C) were also deleted. Following the required data completeness check regarding 3442 study participants at baseline, verification embraced the follow-up data pool. Consequently, 54 (total) cases of missing data on systolic blood pressure (SBP), FBG, high-density lipoproteins (HDL-C), and LDL-C were established. See Figure 1 for details of data verification. 

### 2.2. Anthropometric Measurements

Upon both the first and second assessment, body weight was measured to an accuracy of 0.1 kg using the Tanita S.C.-240 MA body composition analyser. Body height in the upright position and waist circumference were measured with Seca height measure and a metric tape to an accuracy of 0.1 cm, respectively. According to the guidelines of the World Health Organization (WHO), waist circumference (WC) was measured at the midpoint between the top of the iliac crest and the lower margin of the last palpable rib in the mid axillary line (cm), whereas hip circumference was measured at the largest circumference of the buttocks (cm). Both measurements were taken with a metric, stretch-resistant measuring tape, held parallel to the floor at the point of measurement [24]. Body mass index (BMI) was calculated as the quotient of body mass in kilograms and square body height in metres (kg/m^2^). Waist-to-hip ratio (WHR) and waist-to-height ratio (WHTR) were calculated as the quotient of waist circumference and hip circumference, or waist circumference and body height, respectively. Systolic (SBP) and diastolic (DBP) blood pressure was measured twice, making use of a certified Omron blood pressure monitor (Model M3 Intellisense), and a suitably sized cuff. Before taking the measurement, the subject was kept in a relaxed sitting position for 5 min. The first measurement was taken after 5 min, and the second within the following 5 min. The result was then calculated as the arithmetical mean of the two consecutive readings.

### 2.3. Laboratory Measurements

Blood samples were taken in line with standard sampling procedures from a subject in a fasting state, then placed in an insulated box containing several chilled packs, reliably ensuring an internal box temperature of 4 °C, and subsequently forwarded to the Holycross Cancer Center biobank every day at noon. Four samples of different volume were taken (i.e., 10 mL, 5 mL, 4 mL, and 2 mL). The samples of 10 mL and 5 mL were forwarded to the biobank, whereas those of 4 mL and 2 mL were transferred to an on-site lab facility for biochemical testing. The concentrations of FBG, HDL-C, total cholesterol (TC), and triglycerides (TG) were determined in the laboratory against the reference standards, using enzymatic methods. The estimation of LDL-C level was done utilising Friedewald’s equation for TG level under 400 mg/dL. Laboratory tests were carried out with the CB 350i Wiener Lab [19].

### 2.4. Definitions of Outcome Variables 

Changes in SBP, DBP, FBG, HDL-C, LDL-C, and TG were calculated as the differences in their values in the *Healthy Kielce* study, as compared to PONS. In further analyses, the above-referenced outcome variables were treated as the continuous variables. Based on the study by Wing et al. [25], the following criteria of clinically significant improvements in outcome variables were adopted: SBP or DBP reduction by 5 mm/Hg, FBG reduction by 20 mg/dL, HDL-C increase by 5 mg/dL, LDL-C reduction by 10 mg/dL, and TG reduction by 40 mg/dL. 

### 2.5. The Individual Health Status Questionnaire

Smoking status and alcohol consumption were divided into two broad categories, i.e., never (never or former), and current smoker or drinker. Moderate to vigorous physical activity in leisure (MVPA) was calculated against the International Physical Activity Questionnaire (long version). MVPA was calculated based on the number of days when physical activity was pursued, and its duration in leisure time.

### 2.6. Statistical Analyses

Body weight changes were calculated as a percentage change in the *Healthy Kielce* study, as compared to PONS. Based on the continuous variable, the following five categories of body weight changes were subsequently defined, i.e., gained ≥ 3%, stable (gained < 3% or lost < 3%), lost ≥ 3 to < 5%, lost ≥ 5 to < 10%, lost ≥ 10%. Changes in blood pressure, lipids and glycemia due to body weight changes were analysed within the designated categories of those changes. Distribution of the variables under study in the separate categories of body weight changes were analysed using Kruskal-Wallis one-way analysis of variance by ranks and chi-square test. The significance of the differences of outcome variables in the individual categories of body weight changes was assessed by the pairwise comparison test with Benjamini and Hochberg adjustment method. 

Normality of outcome variables was assessed by Shapiro-Wilk test. Since the distribution of the variables under study significantly differed from a normal distribution, the changes in SBP, DBP, FBG, HDL-C, LDL-C, and TG, depending on the adopted categories of body weight changes, were determined through robust regression models. The analyses were adjusted for age, gender, level of outcome variables at baseline, and body weight at baseline. The least square means were plotted, and significance of the differences in outcome variables depending on the category of body weight change were examined using ANOVA test. The significance of the differences between respective study groups was investigated with the aid of the Tukey test. Based on the Poisson regression models with robust standard errors, the associations between weight loss and compliance with the criteria for significantly improved clinical control over CVD risk factors were investigated. Adjusted incident rate ratios (IRRs) and 95% confidence intervals (CIs) were estimated. Covariates for adjusted IRRs comprised: age, gender, smoking history, alcohol drinking, and MVPA status. 

A ten-year absolute risk of fatal CVD in individuals with no atherosclerotic CVD, and changes in this risk owing to changes in body weight, were estimated with an updated SCORE algorithm (Systematic Coronary Risk Evaluation) for Poland. A ten-year risk of fatal CVD was determined based on age, gender, smoking status, SBP, and TC. In compliance with pertinent recommendations of the European Society of Cardiology, the following risk thresholds were adopted, i.e., low risk (SCORE <1%), moderate risk (SCORE ≥1% and <5%), and high to very high risk (SCORE ≥5%) [26]. All cases of self-reported CVD or missing data of CVD (*n* = 717) were excluded from the study database. Ultimately, 2671 (38.5% men) study subjects were covered by the SCORE analysis (Figure 1). *p* values < 0.05 were assumed as statistically significant. Statistical significance is indicated on the graphs by asterisks (* *p* < 0.05; ** *p* < 0.01; *** *p* < 0.001). All statistical analyses were carried out in R (version 3.5.3).

### 2.7. Sensitivity Analysis

A sensitivity analysis was carried out for the associations between the established weight loss categories and compliance with the criteria for significantly improved clinical control over CVD risk factors. All cases (*n* = 1870) with self-reported comorbidities were deleted from the study database (Figure 1). Poisson regression models were then matched using the same set of variables as in the main analysis.

## 3. Results

A population sample of 3388 participants from the PONS and *Healthy Kielce* studies (aged 45–64 years; average age 55.7 years) were assessed, respectively. Men made up 37.9% of the sample. The mean body weight and BMI were 80.6 kg and 29.6 kg/m^2^, respectively. At baseline, no significant differences in body weight were encountered in the designated categories of its changes. Significant differences in BMI in the respective categories of the changes under study resulted from the significant differences in the participants’ height in specific categories. Average values of outcome variables were significantly different, whereas the differences of LDL-C, TC, percentage of participants with BMI ≥ 30 kg/m^2^, and the percentage of drinkers proved non-significant within these categories of change (Table 1).

Table 2. comprises the mean values of the CVD risk factors at baseline, and their changes after a two-year follow-up, in line with the adopted categories of body weight changes. Weight loss by 3% and more was observed in ≈20% of the subjects and was related to the improvement of mean values of the outcome variables. Regretfully, in over 80% of the subjects, no change in body weight whatsoever was encountered, nor body weight gain. In this particular group of subjects, the average values of the CVD risk factors under study indicated a deterioration in individual health status. This was particularly evident in the case of TG.

**Figure 2:**
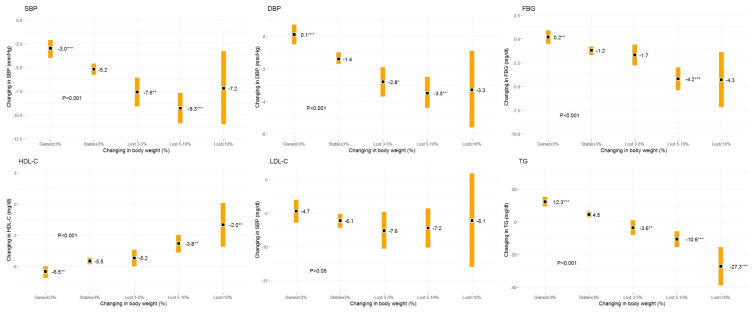
Changes in CVD risk factors by weight loss categories. Notes: Data are presented as the least square means with 95% Cis. Adjustment pertains to age, gender, baseline level of the outcome variables, and baseline weight. Abbreviations: SBP, systolic blood pressure; DBP, diastolic blood pressure; FBG, fasting blood glucose; HDL-C, high-density lipoprotein cholesterol; LDL-C, low-density lipoprotein cholesterol; TG, triglyceride. * *p* < 0.05; ** *p* < 0.01; *** *p* < 0.001 vs. stable category.

Except for LDL-C, changes in CVD risk factors due to weight changes were statistically significant (Figure 2). The least square mean for each one of the risk factors adjusted for age, gender, value of the outcome variable at baseline, and body weight at baseline, improved in each higher category of weight loss. Based on the post hoc test results regarding all factors under study (except LDL-C), significant improvement was noted with regard to weight loss, i.e., 5%–10%. Weight loss of 3%–5% failed to significantly improve the FBG and HDL-C outcome variables, though. In the subjects who lost >10% of body weight the values of the SBP, DBP, and FBG outcome variables did not differ significantly from those of the weight maintenance group. This was probably due to very few cases in this particular category of weight loss. The strongest association between improved control over CVD risk factors and weight loss were observed for TG. Regretfully, in individuals whose body weight increased above 3%, a significant status deterioration regarding CVD risk factors under study was reported (except for LDL-C), as compared to those maintaining stable body weight.

**Figure 3:**
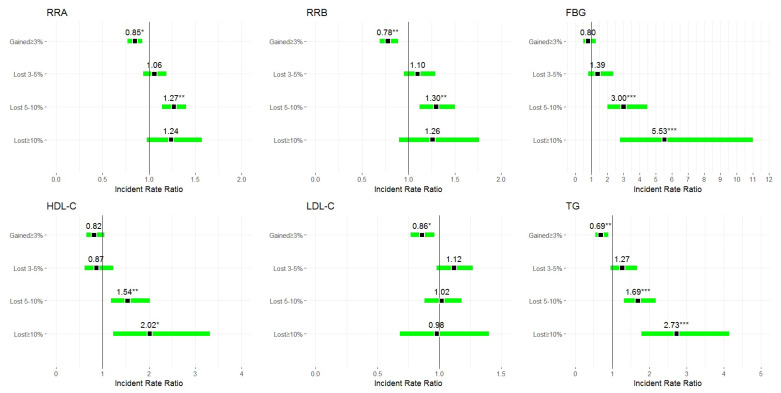
Adjusted incident rate ratios (IRRs) (95% CIs) of clinically significant changes in CVD risk factors associated with pertinent weight change categories. Notes: Adjustment pertains to age, gender, smoking status categorised as non-smoker (never smoker and former smoker) or smoker (current smoker); drinking status categorised as (never drinker and former drinker), or drinker (current drinker); moderate to vigorous physical activity at leisure categorised as yes or no. Abbreviations: SBP, systolic blood pressure; DBP, diastolic blood pressure; FBG, fasting blood glucose; HDL-C, high-density lipoprotein cholesterol; LDL-C, low-density lipoprotein cholesterol; TG, triglyceride. * *p* < 0.05; ** *p* < 0.01; *** *p* < 0.001 vs. stable category.

Appreciably improved clinical control over CVD risk factors was significantly associated with the amount of weight loss (Figure 3). With the exception of LDL-C, the IRRs of outcome variables were higher in the higher weight loss categories. An increase in body weight by 3% or more reduced the IRRs of clinically significant health improvement. After exclusion from the analyses of all cases with self-reported comorbidities, significantly improved clinical control over CVD risk factors (except LDL-C) was mainly related to 5%–10% of weight loss (Table 3). In the other categories of weight changes, the IRR values indicated the associations under study, although of a non-significant character.

The changes in ten-year risk of fatal CVD owing to body weight changes are shown in Table 4. Although these changes proved non-significant across all weight loss categories, any slight weight loss within the 3%–5% and 5%–10% ranges resulted primarily in a reduction of high to very high risk, and in an increase in moderate risk of fatal CVD. Throughout a two-year observation period, sustaining by individuals of just any weight loss was established not to have induced any changes in the low risk of fatal CVD category, whereas weight loss ≥10% was not associated with any changes in the risk of fatal CVD.

## 4. Discussion

In terms of assessing the health consequences induced by changes in body weight, a clear-cut definition of weight maintenance is crucial as a point of reference for any potential changes [27]. Even though weight maintenance can literally mean lack of any weight change whatsoever, human body weight is in fact subject to various changes, even with regard to stable fat and muscle mass. These changes are most often related to daily dietary intake, micturition and defecation, body fluid balance, calibration of the measuring devices, and any attendant measurement bias [28]. A separate issue consists in the actual choice of a unit of measurement which is best suited to addressing weight maintenance effectively, as well as being easy-to-apply to all parties concerned. 

Presently, there are no explicit weight maintenance guidelines in place for adults. Experts highlight the two different, most popular ways of defining weight maintenance after weight loss. The first allows for the likelihood of regaining less than three kg of body weight within two years after previous weight loss and having waist circumference permanently reduced by at least 4 cm [29]. The other does not impose any limits on the body weight which may be regained after previous weight loss, as long as this net loss is sustained for at least one year below 5% of the recommended weight loss, or BMI reduction by 1 unit [30]. The second definition of weight maintenance is supported by the consensus that a 5% weight change is clinically significant. It may therefore be assumed that this is the upper limit of what is termed weight maintenance, yet this may not necessarily be construed as the lower limit. This problem stems from there being no grounds for an arbitrary assumption that, since weight loss of 5% is clinically significant, any weight gain of less than 5% is to be devoid of any clinical significance. Consequently, the present study adopted a more restrictive criterion defining weight maintenance, i.e., a change in body weight of less than ± 3% [28].

Another important issue in assessing any health benefits gained through the treatment of obesity consists in determining the actual magnitude of weight loss which would offer such benefits. It is widely accepted that 5–10% of weight loss is beneficial to one’s health [31,32]. While these assertions are corroborated by the results of numerous studies, it should also be borne in mind that the association between body weight and individual health outcomes is continuous rather than of a threshold-like character [33]. Attempting to establish a cut-off threshold grounded in a significant improvement in one’s health status implies the need to indicate unequivocally what is deemed a significant improvement in individual health status, allowing for the fact that it would be bound to vary for different outcome variables (e.g., blood pressure, lipids, glycemia). 

In one of the first studies addressing the issue of mitigation of the most common complications stemming from obesity, Atkinson proposed three categories of improvement in outcome variables, whilst making use of their reference values [34]. He construed any improvement in a variable under study (e.g., blood pressure, lipids or glucose) as a minimal success. Improvement in study variables by up to 50% within the range of baseline and reference values was deemed an intermediate success, whereas any reduction in the said variables down to the actual reference values was referred to as a full success. At the same time, he highlighted the need for an individually tailored assessment, considering, e.g., age as a confounding factor with regard to the variables under study. Somewhat intuitive (except for full success), although apparently resulting from the author’s own, hands-on experience, categorization of clinical success gave rise to further studies attempting to quantify clinical health improvements attributable to weight loss.

Effective weight loss, when combined with improved clinical control over CVD risk factors, appears to remain in a strong causative association with the type of medical intervention actually applied [35,36]. These may only consist in specific recommendations regarding weight loss, dietary and/or physical activity interventions, and/or medications prescribed, to be pursued in conjunction with regular monitoring of weight changes [37,38,39,40,41]. In extreme cases, surgical interventions leading to dramatic weight loss are also regarded as a viable option, consequently resulting in significantly improved control over the identified CVD risk factors [42]. Differently structured study designs provide discrepant, occasionally conflicting data on the direction and magnitude of the resultant weight changes. In the present study, subjects affected by excessive body weight were only advised to focus on losing weight, whilst remaining in regular consultation with their primary care physician. 

In the present study, weight loss of up to 5–10% was associated with significant improvements in the outcome variables, except for LDL-C, whose changes, regardless of the magnitude of weight loss, were not associated with any health success. Slightly different results were reported by Poobalan et al. [43], based on an extensive review of studies (prospective, cohort studies, and randomized controlled trials), corroborating beneficial effects of long-term (over five years) weight loss, especially in the case of LDL-C. This study indicated a weak association between HDL-C and weight loss, as compared to other lipid levels. According to Walden et al. [44], a significant improvement of LDL-C occurs during the period of reduced caloric intake only. Following the conclusion of a reduction diet, LDL-C increases, despite sustained weight loss. 

The results yielded by this study support the association between modest weight loss (>5%) and a clinically important decrease of SBP and DBP. Regretfully, weight loss ≥ 10% was associated with just any, rather than a clinical improvement in a specific individual health status. Numerous studies have indicated weight loss to be effectively instrumental in decreasing high blood pressure in obese individuals. In the study combined with a four-year follow-up, as conducted by Hasegawa et al. [45] in a population of 2579 Japanese individuals with BMI ≥ 25 kg/m^2^, who were not affected by diabetes mellitus, hypertension or dyslipidaemia, 5% weight loss was associated with normalisation of blood pressure in both genders, even though the volume of this loss was associated with a significant decrease in blood pressure in men only. In the study by Cochrane et al. [46], 26% of modifiable CVD risk was associated with high blood pressure. As a result of the intervention, including weight normalization, a modifiable population risk of CVDs attributed to high blood pressure was decreased by 68%.

In the present study, a modest weight loss (5–10%) was associated with a significant improvement of FBG, and implementation of an improved clinical control over CVD risk factors. Treatment of early symptoms of hyperglycemia, related inter alia to weight loss, is an essential component in reducing the risk of CVDs [12,47,48]. Weight loss may also contribute to an improved control over other cardio-metabolic risk factors in individuals affected by diabetes mellitus (DM). The American Diabetes Association recommends that individuals with the pre-diabetes mellitus condition be referred to an intensive behavioural intervention programme, with a view to losing 7% of their body weight, and then sustaining this reduction, as well as increasing moderate physical activity to a minimum of 150 min/week [49]. The overall body of evidence originating in experimental studies indicates modifications in individual lifestyle as an effective DM risk reduction strategy [50,51,52]. Based on the Diabetes Prevention Programme, the application of lifestyle interventions has reduced the incidence of DM by 58% over three years and resulted in a sustained reduction in the conversion rate by 34% and 27% after 10 and 15 years, respectively [53,54].

Currently applicable CVD prevention guidelines recommend taking preventive action, inter alia, by estimating the absolute CVD risk against the SCORE risk charts. With this in mind, specifically calibrated risk charts have been developed in conformity with the risk factors and mortality levels actually encountered in respective countries [55]. In the present study, body weight loss of 3%–10% was related to the reduction of a 10-year high to very high risk of fatal CVD. Despite these associations having remained non-significant, it is nevertheless worth noting that the interpretation of the results obtained in terms of a population level tends to leave any individually gained health benefits blurred. In the group of study subjects eligible for the SCORE analysis, any weight loss was observed in 18.3% of them. In that group, a 10-year high to very high risk of fatal CVD at baseline was observed in 18.0% of the subjects. Following a two-year follow-up period, weight loss over 3% resulted in a change of fatal CVD risk from high to moderate in 26.1% of the subjects within this group. 

Whilst acknowledging the limitations of the present study, it was highlighted that assessment of the associations between weight loss over 10% and alterations in the variables under study yielded rather dubious results in diagnostic terms. One of the underlying reasons was clearly a considerable (1.4%) underrepresentation of cases with weight loss ≥10%. This consequently affected overall reliability of the estimated variables under study, magnitude of standard errors of these estimates, and very wide 95% confidence intervals. Another potential study limitation consisted in its design. The variables were assessed twice (at baseline and at the end of the follow-up period). Interim body weight measurements pursued within a shorter time interval would have allowed differentiation between maximum weight loss, weight maintenance, and regaining body weight, following its previous maximum loss. Recommendations for weight loss were specific medical recommendations given on a one-off basis to the study subjects at baseline. 

There were no control measures and/or additional motivation offered to the study subjects to follow the guidelines. This resulted in just casual weight loss in ≈18% of the subjects, which does not rule out the likelihood that ineffective health measures may well have been adopted by a much larger percentage of study respondents. A relatively short follow-up period may also have had an impact on the changes in the outcome variables, following changes in individual body weight. The associations under study are usually most manifest in the short term, while later they weaken as a result of body weight having been regained. Hence, not only the actual volume of individual weight loss itself is essential, but also the manner by which any such weight loss will subsequently have been maintained. The study also did not consider any potential risk factors deemed instrumental in weight changes, e.g., uric acid.

## 5. Conclusions

A modest weight loss improves clinical control over CVD risk factors in individuals remaining at risk of being overweight or obese. Weight loss of 5–10% allows clinically significant improvement in blood pressure, HDL-C cholesterol fraction, (but not LDL-C), triglycerides, and fasting glycemia. Since even in the short term a modest weight loss stands to significantly improve individual health outcomes, this method should routinely be recommended as an effective strategy, principally aimed at reducing overall CVD risk. No reported changes or deterioration in body mass variables observed in ≈80% of the study participants highlighted the need for implementation of a series of directive-based, remedial measures, combined with an individually tailored, sustained support programme, in conjunction with systematic monitoring of anticipated individual health outcomes. Indubitably, an assessment procedure for ten-year absolute risk of fatal CVD, facilitating appreciably more accurate identification of patients with high health-loss risk, offers viable support for preventive measures pursued at both individual and population level.

## Figures and Tables

**Table 1:** Basic characteristics of the study group total at baseline, and by the weight change categories.

Variables	Total	Stable	Gained	Lost	Lost	Lost	*p*
	at Baseline	>3% and <3%	≤3%	≥3% and <5%	≥5% and <10%	≥10%	
*n* (%)	3388 (100)	1975 (58.3)	784 (23.1)	310 (9.1)	272 (8.0)	47 (1.4)	<0.001
Sex/men, *n* (%)	1283 (37.9)	800 (40.5)	267 (34.1)	109 (35.2)	98 (36.0)	9 (19.2)	<0.001
Age (years)	55.7 (43.0–64.0)	55.8 (43.0–64.0)	55.0 (43.0–64.0)	56.6 (43.0–64.0)	55.9 (43.0–64.0)	55.6 (45.0–64.0)	<0.001
Height (cm)	164.8 (141.0–198.0)	165.2 (141.0–198.0)	164.6 (145.0–189.5)	164.3 (145.0–190.0)	164.3 (148.0–196.0)	161.5 (148.0–184.0)	<0.01
Weight (kg)	80.6 (53.2–138.0)	80.9 (53.2–138.0)	79.7 (57.4–135.4)	80.1 (57.8–123.8)	81.3 (55.7–120.8)	81.5 (61.5–120.5)	>0.05
BF (%)	34.6 (12.9–57.4)	34.4 (12.9–57.4)	34.6 (14.1–53.7)	35.1 (18.9–53.7)	35.1 (19.3–50.5)	38.6 (20.4–47.8)	<0.001
BMI (kg/m^2^)	29.6 (25.0–52.3)	29.6 (25.0–52.3)	29.4 (25.0–47.2)	29.6 (25.0–46.6)	30.0 (25.0–47.8)	31.2 (25.0–41.5)	<0.01
WC (cm)	95.2 (64.0–142)	95.5 (68.0–135.0)	94.2 (70.0–142)	95.0 (71.0–140.0)	95.7 (73.0–133.0)	96.3 (64.0–118.0)	<0.05
WHR	0.9 (0.6–1.7)	0.9 (0.6–1.7)	0.9 (0.7–1.2)	0.9 (0.7–1.2)	0.9 (0.7–1.2)	0.9 (0.7–1.0)	<0.05
WHTR	0.6 (0.4–0.9)	0.6 (0.4–0.8)	0.6 (0.5–0.8)	0.6 (0.4–0.9)	0.6 (0.5–0.8)	0.6 (0.4–0.8)	<0.01
SBP (mm/Hg)	139.5 (92.5–230.0)	140 (92.5–230.0)	137.6 (93.5–207.0)	138.6 (103.5–203.5)	141.7 (98.0–214.0)	144.3 (96.5–224.0)	<0.01
DBP (mm/Hg)	82.5 (50.0–136.5)	82.9 (50.0–136.5)	81.5 (57.5–123.0)	81.8 (54.5–133.0)	83.2 (56.5–114.5)	82.3 (58.5–106.0)	<0.01
FBG (mg/dL)	99.5 (66.1–358.0)	99.5 (70.0–358.0)	98.1 (66.1–256.0)	98.9 (72.0–219.0)	102.5 (68.0–310.0)	107.7 (75.0–238.0)	<0.05
HDL-C (mg/dL)	56.5 (20.0–134.0)	56.1 (20.2–120.0)	57.6 (27.0–134.0)	55.7 (20.0–98.0)	56.6 (27.2–104.0)	60.8 (28.0–100.0)	<0.05
LDL-C (mg/dL)	127.1 (35.4–277.4)	128.0 (41.2–277.4)	124.2 (35.4–252.2)	130.5 (52.6–211.6)	125.6 (39.8–260.8)	122.9 (64.7–179.2)	>0.05
TC (mg/dL)	208.1 (98.0–361.0)	209.1 (100.0–360.0)	204.8 (98.0–353.0)	210.4 (103.0–316.0)	206.6 (110.0–361.0)	209.9 (142.9–290.0)	>0.05
TG (mg/dL)	122.2 (28.0–397.0)	125.2 (28.0–389.0)	114.5 (36.0–353.0)	121.3 (37.0–397.0)	122.1 (34.0–371.0)	130.6 (52.0–338.0)	<0.001
BMI ≥ 30, *n* (%)	1248 (36.8)	732 (37.1)	270 (34.4)	113 (36.5)	109 (40.1)	24 (51.1)	>0.5
Smoker, *n* (%)	483 (14.3)	267 (13.5)	137 (17.5)	44 (14.2)	29 (10.7)	6 (12.8)	<0.05
Drinker, *n* (%)	2912 (86.0)	1714 (86.8)	667 (85.1)	266 (85.8)	227 (83.5)	38 (80.9)	>0.05
MVPA, *n* (%)	1062 (31.4)	653 (33.1)	234 (29.9)	94 (30.3)	73 (26.8)	8 (17.0)	<0.05

Notes: Data are presented as mean (range), unless stated otherwise. *p* is statistically significant at alpha level of 0.05. Abbreviations: BF, body fat; BMI, body mass index; WC, waist circumference; WHR, waist-to-hip ratio; WHTR, waist-to-height ratio; SBP, systolic blood pressure; DBP, diastolic blood pressure; FBG, fasting blood glucose; HDL-C, high-density lipoprotein cholesterol; LDL-C, low-density lipoprotein cholesterol; TC, total cholesterol; TG, triglyceride; MVPA, moderate to vigorous physical activity in leisure.

**Table 2:** Changes in CVD risk factors from baseline to two-year follow-up, stratified by the weight change categories.

Variables	Total at Baseline	Stable	Gained	Lost	Lost	Lost
		>−3% and <3%	≥3%	≥3% and <5%	≥5% and <10%	≥10%
*n* (%)	3388 (100)	1975 (58.3)	784 (23.1)	310 (9.1)	272 (8.0)	47 (1.4)
SBP (mm/Hg)	139.5 ± 18.9	−4.9 ± 16.0	−2.1 ± 16.2 ***	−6.9 ± 16.0	−9.7 ± 16.3 ***	−9.5 ± 17.3
DBP (mm/Hg)	82.5 ± 10.2	1.5 ± 9.1	0.4 ± 9.4 ***	−2.5 ± 10.3	−3.8 ± 9.4 ***	−3.1 ± 10.0
FBG (mg/dL)	99.5 ± 19.4	−0.4 ± 16.3	1.5 ± 14.0 **	−1.1 ± 16.8	−4.4 ± 18.7 ***	−9.4 ± 25.1 **
HDL-C (mg/dL)	56.5 ± 13.7	−4.7 ± 8.8	−6.1 ± 9.5 **	−4.2 ± 8.0	−2.8 ± 10.4 **	−2.3 ± 11.4
LDL-C (mg/dL)	127.1 ± 33.6	−6.7 ± 31.7	−3.4 ± 30.5 **	−8.6 ± 29.0	−6.3 ± 31.0	−2.5 ± 29.3
TG (mg/dL)	122.2 ± 58.1	7.9 ± 54.4	21.5 ± 52.0 ***	−1.4 ± 47.1 ***	−5.3 ± 55.5 ***	−30.9 ± 58.8 ***

Notes: Data are presented as mean ± standard deviation, unless stated otherwise. Abbreviations: SBP, systolic blood pressure; DBP, diastolic blood pressure; FBG, fasting blood glucose; HDL-C, high-density lipoprotein cholesterol; LDL-C, low-density lipoprotein cholesterol; TG, triglyceride. * *p* < 0.05; ** *p* < 0.01; *** *p* < 0.001 vs. stable category.

**Table 3:** Adjusted IRRs (95% CIs) of clinically significant changes in CVD risk factors associated with the weight change categories.

Variables	Gained ≥3%	Stable >−3% and <3%	Lost ≥3 to <5%	Lost ≥5 to <10%	Lost ≥10%
SBP 5 mm/Hg decrease	0.81 (0.70, 0.93) *	1 (ref)	0.99 (0.83, 1.18)	1.40 (1.22, 1.60) **	1.15 (0.74, 1.77)
DBP 5 mm/Hg decrease	0.74 (0.60, 0.90) *	1 (ref)	1.21 (0.97, 1.51)	1.45 (1.18, 1.78) *	1.27 (0.72, 2.24)
FBG 20 mg/dL decrease	1.60 (0.82, 3.10)	1 (ref)	2.19 (0.99, 4.83)	3.34 (1.64, 6.79) **	2.58 (0.34, 19.71)
HDL-C 5 mg/dL increase	0.80 (0.56, 1.15)	1 (ref)	1.11 (0.70, 1.78)	1.99 (1.37, 2.91) **	2.11 (0.89, 5.01)
LDL-C 10 mg/dL decrease	0.80 (0.67, 0.95) *	1 (ref)	1.07 (0.88, 1.31)	1.04 (0.83, 1.29)	1.29 (0.80, 2.10)
TGC 40 mg/dL decrease	0.73 (0.50, 1.06)	1 (ref)	1.56 (1.08, 2.25) *	1.80 (1.23, 2.64) **	2.11 (0.85, 5.25)

Abbreviations: SBP, systolic blood pressure; DBP, diastolic blood pressure; FBG, fasting blood glucose; HDL-C, high-density lipoprotein cholesterol; LDL-C, low-density lipoprotein cholesterol; TG, triglyceride. * *p* < 0.05; ** *p* < 0.01; vs. stable category.

**Table 4:** Changes in ten-year absolute risk of fatal CVD from baseline to two-year follow-up, as stratified by Systematic Coronary Risk Evaluation (SCORE) and weight change categories, respectively.

Absolute	Total	Stable	Gained	Lost	Lost	Lost	*p*
Risk		>−3% and <3%	≥3%	≥3% and <5%	≥5% and <10%	≥10%	
*Baseline*							<0.001
Low	223 (8.3)	127 (8.2)	66 (10.4)	14 (5.8)	14 (6.6)	2 (5.9)	
Moderate	1859 (69.6)	1035 (66.9)	454 (71.4)	176 (72.4)	165 (78.2)	29 (85.3)	
High to very high	589 (22.1)	385 (24.9)	116 (18.2)	53 (21.8)	32 (15.2)	3 (8.8)	
*Follow-up*							<0.001
Low	226 (8.5)	127 (8.2)	71 (11.2)	13 (5.4)	13 (6.2)	2 (5.9)	
Moderate	1949 (73.0) **	1101 (71.2) **	458 (72.0)	186 (76.5)	174 (82.5)	30 (88.2)	
High to very high	496 (18.6) **	319 (20.6) **	107 (16.8) *	44 (18.1)	24 (11.4)	2 (5.9)	

Notes: Data are presented as *n* (%). Abbreviations: * *p* < 0.05; ** *p* < 0.01 vs the corresponding category at baseline.
